# Persistence of markers of chloroquine resistance among *P. falciparum* isolates recovered from two Nigerian communities

**DOI:** 10.5281/zenodo.10878545

**Published:** 2014-02-26

**Authors:** Yetunde A. Olukosi, Muyiwa K. Oyebola, Olusola Ajibaye, Bassey A. Orok, Olugbenga O. Aina, Chimere O. Agomo, Bamidele A. Iwalokun, Samuel K. Akindele, Veronica N.V. Enya, Hilary I. Okoh

**Affiliations:** 1 Malaria Research Laboratory, Nigerian Institute of Medical Research, 6 Edmond Crescent, P.M.B. 2013, Yaba, Lagos, Nigeria; 2 Parasitology and Bioinformatics Unit, Faculty of Science, University of Lagos, Lagos, Nigeria

## Abstract

**Background:**

A recovery in chloroquine efficacy following a period of cessation has raised the possibility of its reintroduction for malaria chemotherapy. We investigated the prevalence of the major markers of chloroquine resistance years after the withdrawal of the drug in Nigeria.

**Materials and Methods:**

Finger prick blood samples were collected from participants presenting with symptoms of malaria in two selected health centres each representing Lekki and Ijede communities of Lagos, Nigeria. Thick and thin blood smears were prepared for microscopy and dry blood spots made from malaria-positive participants for parasite DNA extraction. The detection of mutations in the *Plasmodium falciparum* chloroquine resistance transporter (*pfcrt)* and *P. falciparum* multidrug resistance (*pfmdr1)* genes was performed by nested polymerase chain reaction (PCR) and restriction fragment length polymorphism (RFLP).

**Results:**

Of the 1527 blood samples that were confirmed by PCR to be *P. falciparum* positive, 412 and 344 were typed for the molecular detection of *pfcrt* and *pfmdr1* gene mutations, respectively. The mutant alleles of *pfcrt* were present among 290 (70%) parasite carriers while the *pfmdr1* mutant allele was found in 117 (34%) of the total population. There were higher distributions of the mutant alleles for the two loci in Ijede than in Lekki. The observed frequencies of *pfcrt* mutant alleles in the two parasite populations were in agreement with the expected frequencies predicted by Hardy-Weinberg. In comparing data with studies conducted between 2000 and 2002 in Ijede, we observed an increase in the prevalence of mutant type *pfcrt* against a marginal decline in the *pfmdr1* mutant type.

**Conclusion:**

The high frequencies of *pfcrt* mutation are suggestive of a persistent drug pressure and continuing inefficacy of chloroquine as an antimalarial drug.

## 1 Introduction

Antimalarial drug resistance has emerged as one of the greatest challenges facing malaria control. The emergence of drug-resistant parasites over the last few decades has affected the epidemiology of malaria and options for its treatment [1], and now there is evidence of decreased sensitivity to the artemisinins [2-4]. Chloroquine (CQ) was previously used in the treatment of malaria until it was no longer the drug of choice in most malaria-endemic countries as a result of the emergence and spread of resistant parasite strains [5]. Resistance to CQ is primarily mediated by a replacement of lysine by threonine at codon 76 (K76T) amidst other ancillary mutations [6]. An allele encoding tyrosine at codon 86 (N86Y) of the *pfmdr1* gene on chromosome 5 has also been associated with CQ resistance [7]. In response to high rates of treatment failure with CQ, Nigeria’s Ministry of Health replaced CQ with artemisinin-based combination therapy for the treatment of falciparum malaria in 2005 [8]. A previous report on CQ efficacy tracking has shown a phenomenal reduction in the prevalence of the major marker associated with CQ-resistant falciparum malaria years after the withdrawal of the drug, indicating resurged efficacy [9]. This reversal raised the possibility of re-introducing this cheap drug for malaria treatment in Africa. In Nigeria, since the withdrawal of CQ, and the subsequent introduction of combination therapies, there has been insufficient information on the distribution of the markers of CQ resistance. We assessed the prevalence of molecular markers associated with CQ resistance in order to determine whether CQ resistance has waned following the cessation of its use.

## 2 Materials and Methods

### 2.1 Study areas and design

The study was conducted in two healthcare centres within Lekki and Ijede communities in Lagos, Nigeria. A retrospective analysis was carried out using blood spots collected from patients during clinical trials of CQ conducted in 2002. Samples collected from infected patients in 2010 during parasite diversity investigations were also analysed. Written consent was obtained from participants or guardians and assent in cases where participants were children.

### 2.2 Sampling and malaria microscopy

Finger prick blood samples were collected from which thick and thin blood films were prepared on microscope slides. The slides were then stained with 10% Giemsa and the thick films were examined for the presence of malaria parasites. Parasitaemia was determined from the thin films by counting the asexual stages of the parasite against 200-500 leucocytes followed by multiplication by 8000. A minimum of 2-200 high-power fields (HPF) were examined depending on parasitaemia levels, with 200 HPF being examined before samples were declared negative for parasites [10]. All positive blood samples were spotted on 3MM Whatman® filter paper (Whatman International Ltd., Maidstone, England), air-dried and stored in plastic bags with silica gel at ambient temperature and then transported to the Malaria Research Laboratory, Nigerian Institute of Medical Research, Lagos for molecular analyses. Ethical approval was obtained from the Institutional Review Board of the Nigerian Institute of Medical Research, Yaba, Lagos as well as the management of the study health centres. Treatment of participants that were *P. falciparum*-positive was carried out following standard practices of the health facilities.

### 2.3 Parasite DNA extraction

Parasite DNA was extracted from filter paper blood spots and molecular detection of *P. falciparum* was carried out with slight modifications of the methods as previously described [11].

### 2.4 Molecular detection of mutations in drug targets

The detection of mutations responsible for CQ resistance was performed by amplifying sequences marking the *pfcrt* and *pfmdr1* genes using nested PCR followed by restriction fragment length polymorphism (RFLP) according to previously described procedures [12]. Primers used for *pfcrt* K76T primary amplification included Crtp1 and Crtp2 while the secondary PCR was conducted by using the forward primer Crtp3 and the reverse primer Crtp4 (Table 1). After amplification, 20 μl of the amplicons was incubated overnight at 50°C with mutation-specific restriction enzyme *Apo I*. In the PCR products, the DNA sequence was cleaved at the wild-type codon site (if present) into two fragments (98 and 72 bp), while the mutant allele was not cut (170 bp). The digested products were separated by electrophoresis in a 2% agarose gel containing ethidium bromide, and DNA was visualised by ultraviolet transillumination. Similarly, amplification of codon 86 of the *pfmdr1* gene was carried out using the following primers: Mdr1 and Mdr2 for the primary PCR reactions and Mdr3 and Mdr4 for the secondary reactions after which restriction with *ApoI* was done. DNA fragments were compared by size and with the PCR products generated from genomic DNA of the 3D7 and Dd2 strains (used as references for susceptible and resistant genotypes, respectively).

**Table 1:** PCR primer sequences for amplification of codon 76 of *P. falciparum* chloroquine resistance transporter gene

Primer pairs	Sequence	Cycling conditions	
Crtp1 (forward primer)	5′-CCGTTAATAATAAATACACGCAG-3′	94°C for 3 mins	}x45
Crtp2 (reverse primer)	5′-CGGATGTTACAAAACTATAGTTACC-3′	94°C for 30 secs	
Crtd1 (forward primer)	5′-TGTGCTCATGTGTTTAAACTT-3′	56°C for 30 secs	
Crtd2 (reverse primer)	5′-CAAAACTATAGTTACCAATTTTG-3′	60°C for 1 min	
		Hold at 4°C	
Mdr1 (forward primer)	5′-GCGCGCGTTGAACAAAAAGAGTACCGCGTG-3′	95°C for 5 mins	
Mdr2 (reverse primer)	5′-GGGCCCTCGTACCAATTCCTGAACTCAC-3′	95°C for 30 secs	}x45
Mdr3 (forward primer)	5′-TTTACCGTTTAAATGTTTACCTGC-3′	45°C for 30 secs	
Mdr4 (reverse primer)	5′-CCATCTTGATAAAAAACACTTCTT-3′	65°C for 45 secs	
		72°C for 5 mins	
		Hold at 4°C	

### 2.5 Statistical analysis

Frequencies of mutations at *pfcrt*-76, *pfmdr1*–86 alleles were calculated as the proportion of samples carrying the mutant form out of the total of all samples carrying either only the mutant form or only the wild-type form. Chi-square tests were applied to compare the temporal distribution of CQ-resistant alleles.

## 3 Results

A total of 1185 patients were screened in 2002 while 1818 individuals were screened in 2010. A total of 3003 febrile patients were screened in both years (2013 at Lekki and 990 at Ijede) out of which microscopy detected 1655 (55.1%) malaria-infected individuals. Of the 1527 blood samples confirmed to be *P. falciparum* positive by species -specific PCR, 412 samples were typed for the molecular detection of *pfcrt* mutation (150 samples from 2002 archives and 262 collected in 2010) whereas 344 samples (150 from 2002 archives and 194 from the 2010 samples) were typed for *pfmdr1* mutation.

**Figure 1.**
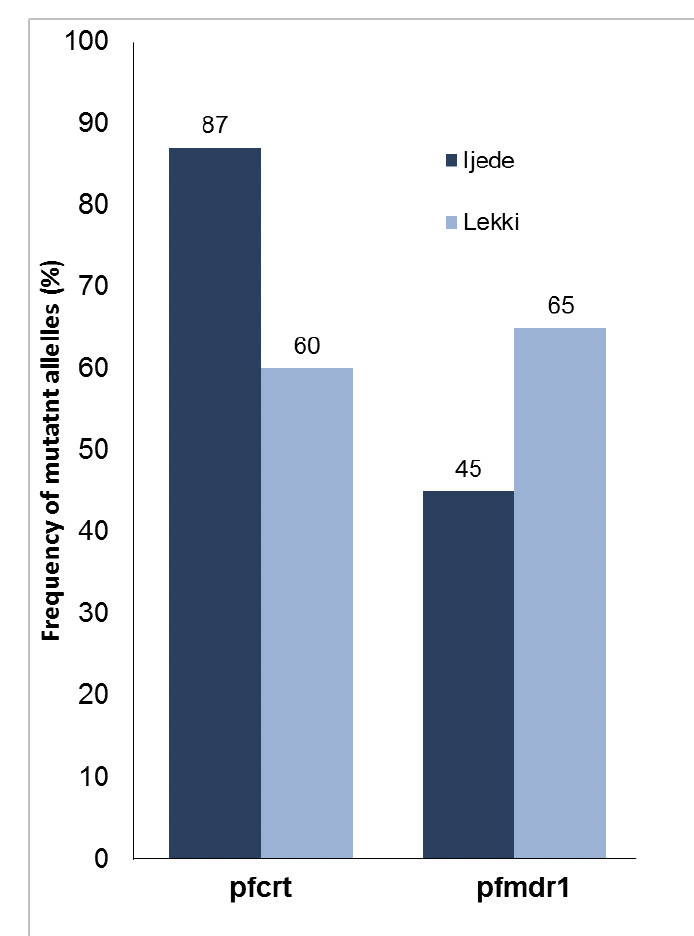
Frequency of mutant alleles in the *Plasmodium falciparum* chloroquine resistance transporter gene (*pfcrt*; A) and multi-drug resistance gene (*pfmdr1*; B) in samples collected in 2010.

**Figure 2.**
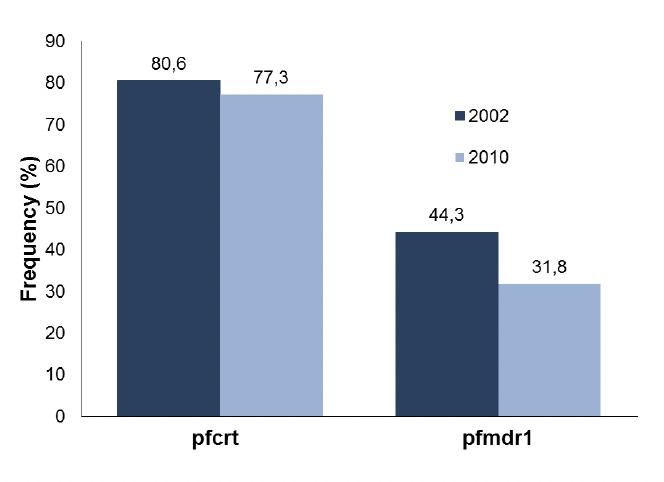
Pre (2002) and post (2010) chloroquine proscription frequencies of *pfcrt* and *pfmdr*1 mutant alleles.

When all parasite samples were taken into account, the *pfcrt* mutant allele was present in 290 (70.4%) *P. falciparum* samples whilst *pfmdr1* 86Y was detected in 117 (34.0%) individuals. The proportion of the *pfcrt* 76T allele in Ijede was higher than in Lekki whilst the reverse was the case for the *pfmdr* 86Y allele (Figure 1). There were higher frequencies of the mutant alleles for both *pfcrt*-76T and *pfmdr*-86Y loci among the 2002 parasites than in the 2010 population. (Figure 2). However, these disparities were not significant (P = 0.836).

## 4 Discussion

It has been documented that the removal of drug pressure exposes resistant parasites to increased competition leading to a decline in the frequency of resistance conferring mutations [13], as reported in Malawi where after just a decade of non-use CQ cleared 100% of asymptomatic *P. falciparum* infections [14]. Similarly, the prevalence of mutant alleles of *pfcrt* in coastal Tanzania decreased after only two and half years of CQ withdrawal [15]. The results of the present study show that the frequency of resistance alleles did not reduce significantly years after CQ use had been stopped. This was consistent with the observation of high *pfcrt* K76T mutations in western Kenya years after CQ withdrawal [16]. The high level distribution of the resistance markers observed in this study supports the previous report that CQ remained widely used at the community level even five years after its withdrawal [17]. This may have been caused by poor educational programmes for rural drug retailers during the change-over period, which led to low proportions of drug users purchasing adequate doses of the ACT first-line drugs.

Alternatively, the use of amodiaquine (AQ), a compound belonging to the group of 4-aminoquinolines that is also structurally similar to CQ, in the newly introduced combination therapy may as well maintain some selection pressure for CQ-resistant parasites. Association studies [18], showing a cross-resistance between CQ and AQ may suggest that the use of artesunate plus amodiaquine contributes to the continued persistence of the mutant *pfcrt* genotype.

Although a marginal decline in the frequency of mutant *pfmdr1*86Y allele was reported in the parasite populations, this may not be sufficient evidence of gradual reversal to CQ susceptibility as the mutation is by itself insufficient to confer resistance [19]. However, on account of reports of the association of tolerance/resistance of *P. falciparum* parasites to artemether/lumefantrine (A/L), the most commonly used ACT in the country, with the selection of susceptible *pfmdr1* N86 allele [20-22], an increase in the distribution of the wildtype allele may point to impending A/L insensitivity in the country.

## 5 Conclusion

The high frequency of the K76T *pfcrt* mutation in contemporary infections suggests that CQ resistance has persisted despite its withdrawal in Nigeria. Continued surveillance of this mutation will be useful to check whether resistance has decreased significantly.
